# Synergistic anti-osteoporosis effects of *Anemarrhena asphodeloides* bunge–Phellodendron chinense C.K. Schneid herb pair via ferroptosis suppression in ovariectomized mice

**DOI:** 10.3389/fphar.2024.1378634

**Published:** 2024-10-24

**Authors:** Xuehui Deng, Wenlong Xiao, Bingfeng Lin, Fang Wang, Li Song, Nani Wang

**Affiliations:** ^1^ Department of Medicine, Zhejiang Academy of Traditional Chinese Medicine, Hangzhou, China; ^2^ School of Pharmacy, Zhejiang Chinese Medical University, Hangzhou, China; ^3^ School of Pharmacy, Hangzhou Medical College, Hangzhou, China

**Keywords:** *Anemarrhena asphodeloides* bunge, *Phellodendron* chinense C.K. Schneid, herb pair, osteoporosis, ferroptosis

## Abstract

**Introduction:**

Ferroptosis plays a crucial role in the progression of postmenopausal osteoporosis. *Anemarrhena asphodeloides* Bunge/*Phellodendron chinense* C.K. Schneid (AA/PC) is the core herb pair in traditional Chinese medicines formulae for postmenopausal osteoporosis treatment. However, the synergistic effects, and mechanisms, of AA/PC on alleviating ferroptosis and postmenopausal osteoporosis remain unclear.

**Methods:**

The goal herein was to analyze the effective ingredients and molecular mechanisms of AA/PC in the treatment of osteoporosis through serum pharmacochemistry, network pharmacology, metabolomics analysis, and pharmacodynamics evaluation. A bilateral ovariectomized (OVX) mouse model was established.

**Results and Discussion:**

Micron-scale computed tomography analysis showed that AA/PC increased bone mineral density in OVX mice. The effects of AA/PC were better than AA or PC alone on inhibiting the bone resorption marker nuclear factor of activated T-cells 1. Furthermore, five absorbable compounds were detected in serum: mangiferin, magnoflorine, berberine, timosaponin BIII, and timosaponin AIII. Network pharmacology showed these compounds had close relationship with seven ferroptosis targets. Importantly, compared with AA or PC alone, the AA/PC herb pair exerted better effects on regulating crucial ferroptosis pathways, including the system xc-/glutathione/glutathione peroxidase 4, transferrin receptor/ferritin, and acyl-CoA synthetase long chain family member 4/polyunsaturated fatty acids signaling pathways. These results indicate that AA/PC exerts synergistic effects on regulating glutathione synthesis, iron homeostasis, and lipid metabolism in ferroptosis. This work lays the foundation for further development and use of AA/PC herb pair for preventing and treating postmenopausal osteoporosis.

## Graphical Abstract

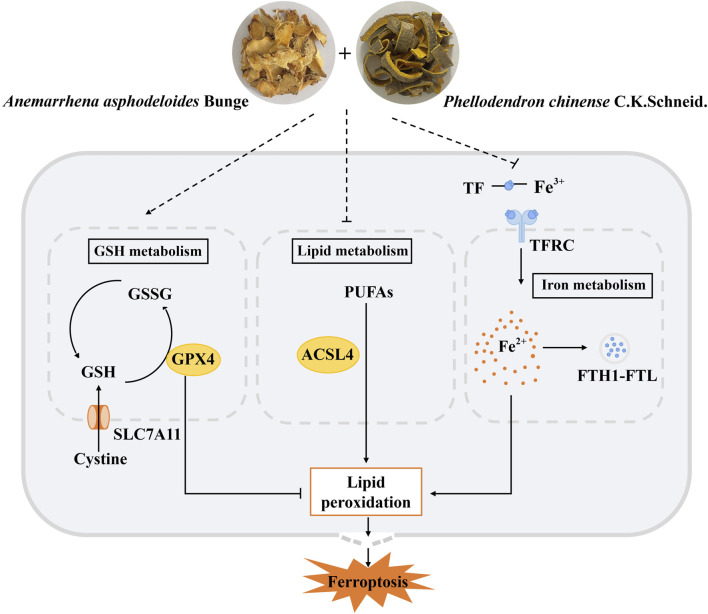

## 1 Introduction

Postmenopausal osteoporosis is characterized by destructed bone, reduced bone mineral density (BMD), and increased bone fragility (Yang et al., 2021). Approximately 50% of postmenopausal women are affected by osteoporosis (Li et al., 2020a). Increasing evidence shows that ferroptosis is involved in the occurrence and development of osteoporosis, and that its inhibition can effectively prevent osteoporosis (Gao et al., 2022). Clinical studies have shown that increased total body iron stores could be an independent risk factor for accelerated bone loss in postmenopausal women (Kim B. J. et al., 2012). Ferroptosis is caused by iron-dependent lipid peroxidation and the accumulation of reactive oxygen species. Mechanically, ferroptosis is associated with iron metabolism disorder, lipid peroxidation accumulation, and glutathione (GSH) and solute carrier family 7 member 11 (SLC7A11) deficiency (Yuan et al., 2022). Among the multiple regulators of ferroptosis, the SLC7A11/GSH/glutathione peroxidase 4 (GPX4) pathway is the main ferroptosis-suppressing pathway. SLC7A11, a transmembrane protein, imports extracellular cystine for GSH synthesis. GSH acts as a cofactor for GPX4, which is a major enzyme catalyzing the reduction of phospholipid hydroperoxides (Chen X. et al., 2021). Ferroptosis is also regulated by the iron metabolism pathway, which involves transferrin receptor protein 1 (TFRC), transferrin (TF), ferritin heavy chain (FTH) and light chain (FTL). In addition to the reduced GSH synthesis and altered iron homeostasis, lipid metabolism is closely related to ferroptosis. Free polyunsaturated fatty acids (PUFAs) can be incorporated into cell membrane by Acy-CoA synthetase long-chain family member 4 (ACSL4) and undergo lipid peroxidation through enzymatic and non-enzymatic pathways (Li et al., 2020b). Increasing evidence has confirmed that progression of postmenopausal osteoporosis is always accompanied by impaired iron homeostasis and elevated lipid peroxidation (Yan et al., 2022). Thus, ferroptosis has been considered a potential therapeutic target for postmenopausal osteoporosis.

Herb pairs act as a basic composition unit of Chinese medicine formulas (Hu X. et al., 2022). *Anemarrhena asphodeloides* Bunge (AA) and *Phellodendron chinense* C.K. Schneid (PC) are a commonly used herb pair in clinical treatments of osteoporosis (Shi et al., 2022), including Zishen pill, Zhibai Dihuang pill, and Dabuyin pill. Our previous work showed that when the two drugs are combined as an herb pair at a 1:1 ratio, AA/PC possesses anti-diabetic osteoporotic effects in streptozotocin-induced rats (Xu et al., 2022a). Increasing studies also show that several components of AA/PC—including berberine, timosaponin AIII, and timosaponin BII (Lee et al., 2008; Wang et al., 2020; Wang L. et al., 2021)—have protective effects on osteoblasts (Wang N. et al., 2021; Wang et al., 2020; Chen Q. C. et al., 2021; Lin et al., 2023). However, the synergistic effects of AA/PC on postmenopausal osteoporosis remain to be clarified.

Herein, we explored the synergistic anti-osteoporosis effects of AA/PC on a bilateral ovariectomy (OVX)-induced mouse model. Serum pharmacochemistry was used to detect the absorbable components of AA/PC. Thus, a network pharmacology approach was developed to discover the relations among absorbable component targets and ferroptosis-related genes. Experimental validation was carried out to identify the synergetic mechanisms of AA/PC as the SLC7A11/GSH/GPX4, TF/ferritin, and ACSL4/PUFAs signaling pathways. To our knowledge, this study is the first to assess the combination mechanisms of this herb pair for its anti-osteoporosis and anti-ferroptosis effects. These results will provide a method for clarifying the combined mechanisms of AA/PC for treating postmenopausal osteoporosis.

## 2 Materials and methods

### 2.1 Plant materials

Crude AA and PC were collected from Zhejiang Jingyuetang Pharmaceutical Co. Ltd (Shaoxing, Zhejiang, China). The voucher specimens of AA (No. RA2019090821) and PC (No. PA2019052101) were deposited at the Department of Medicine, Zhejiang Academy of Traditional Chinese Medicine, Zhejiang, China. AA and PC are prescribed in a ratio of 1:1 (w/w) (Xu et al., 2022b). The plant name was verified at https://www.worldfloraonline.org. The hot water extraction method was used to prepare the drugs (Wu et al., 2019). Briefly, the AA and PC mixture was extracted with water at a ratio of 1:10 (w/v) for 2 h at 100°C. After filtration, the residue was extracted again. The filtrates were combined and lyophilized with a freezing dryer system (SCIENTZ-12N/A, Ningbo Scientz Co. Ltd. Ningbo, Zhejiang, China) (Shen et al., 2022).

### 2.2 Experimental animals and treatment

All animal experiment protocols herein were approved by the Animal Care and Use Committee of the Zhejiang Academy of Traditional Chinese Medicine (Approval No. ZATCM 2022-030) and conformed to the National Research Council’s Guide for the Care and Use of Laboratory Animals. Female C57BL/6 mice (20–22 g) were purchased from Hangzhou Medicine College (Hangzhou, Zhejiang, China) and housed at 25°C with a 12-h light/dark cycle, with free access to food and water. Female mice were randomly divided into a Sham group and four OVX groups (n = 5/group). OVX mice had ovaries removed through a back flank incision (Ali et al., 2022). Sham group mice had adipose tissue removed, without ovary removal. For ovariectomy, mice were anesthetized, and a 5 mm back incision was made; ovaries on both sides were exposed after removing the muscle tissues; ovaries were then removed after tubal ligation, and the wound was sutured. OVX mice were orally dosed with vehicle (Phosphate buffer saline, PBS, Mod group), AA (7 g/kg/d, AA group), PC (7 g/kg/d, PC group), or AA/PC aqueous extract (7 g/kg/d, AA/PC group) for 12 weeks. The Sham group was administered gavage with vehicle (PBS) for 12 weeks. Dosages were based on previous reports (Xu et al., 2022a; Zhao et al., 2018). 3 months later, the mice were anesthetized and sacrificed after blood collection, by eyeball enucleation. The experimental diagram was shown in Supplementary Figure S1.

### 2.3 Micro-computed tomography (micro-CT) analysis

After sacrifice, the femurs of mice from each group were fixed and scanned by micro-CT (Skyscan 1172, Bruker, Belgium). Osteoporosis-related bone indexes, including BMD, are based on micro-CT (Zhang et al., 2018).

### 2.4 Immunohistochemistry (IHC)

IHC analysis was consistent with previous reports (Xia et al., 2020). Femurs were collected, fixed with 4% formalin for 48 h, decalcified in 10% ethylenediamine tetra acetic acid, and paraffin-embedded. Sections were incubated with primary antibodies against nuclear factor of activated T-cells 1 (NFATc1, #SC7294, Santa Cruz Biotechnology, CA, United States, 1:2000), SLC7A11 (#BM5318, Boster, CA, United States, 1:2000), GPX4 (#DF6701, Affinity, Changzhou, China, 1:2000), TF receptor (TFRC, #AF5343, Affinity, 1:2000), TF (#17435-1-AP, Proteintech, Wuhan, China, 1:2000), FTH1 (#DF6278, Affinity, 1:2000), FTL (#10727-1-AP, Proteintech, 1:2000), 4-hydroxynonenal (4-HNE, #ab46545, Abcam, MA, United States, 1:2000), and ACSL4 (#DF12141, Affinity, 1:2000).

### 2.5 Ultra-high performance liquid chromatography combined with quadrupole time-of-flight mass spectrometry (UPLC-QTOF-MS) analysis

The herb extract and drug-containing serum were analyzed using UPLC-QTOF-MS (Ma et al., 2021). For serum analysis, 100 μL of sample was added to 300 μL methanol to precipitate the protein (Michopoulos et al., 2009). The mixture was then vortexed for 30 s and centrifuged at 12,000 rpm and 4 °C for 30 min. The supernatant was then transferred and lyophilized. The resulting residue was combined with 200 μL 10% acetonitrile, and centrifuged at 12,000 rpm for 10 min. After microfiltration, 3 μL of each supernatant sample was injected and analyzed (Wang L. et al., 2021).

Analysis experiments were performed using a UPLC system (Waters ACQUITY I-Class Plus, Waters, Framingham, MA, United States) equipped with a mass spectrometer (SCIEX X-500R Q-TOF, AB SCIEX, Framingham, MA, United States). The mobile phase was composed of water +0.1% formic acid (A) and acetonitrile (B) at a flow rate of 0.3 mL/min. The gradient elution was as follows: 0–5 min, 10%–30% B; 5–10 min, 30%–45% B; 10–13 min, 45%–90% B; 13–15 min, 90%–100% B. The mass spectrometer was operated in positive and negative ion modes. The analysis parameter was as follows: ion source temperature of 600°C; collision energy of 35 V; and declustering potential of 60 V; Mass ranges for TOF-MS was m/z 50–1500. SCIEX OS software (AB SCIEX) was used to record and process data.

### 2.6 Network pharmacology

Network pharmacology was performed as previously described (Zhu et al., 2021). The chemical structure and simplified molecular input line entry specification (SMILES) of AA/PC was obtained from PubChem (https://pubchem.ncbi.nlm.nih.gov/) (O’Boyle, 2012). Target prediction of AA/PC was carried out using SwissTargetPrediction (http://www.swisstargetprediction.ch/) (Aihaiti et al., 2021), with species limited to *“Homo sapiens”*. The protein-protein interaction (PPI) network of target genes was obtained from the Search Tool for the Retrieval of Interacting Genes/Proteins database (STRING 11.0; https://cn.string-db.org/) (Szklarczyk et al., 2021). The PPI network was visualized using Cystoscape v3.10.0. In the PPI network, nodes represent the target proteins, while edges represent the predicted or validated interaction between proteins (Xiang et al., 2021). Kyoto encyclopedia of genes and genomes (KEGG) pathway analysis and gene ontology (GO) enrichment analysis were conducted using the core targets in the DAVID database (https://david.ncifcrf.gov/) (Ke et al., 2019).

### 2.7 Quantitative real-time reverse transcription polymerase chain reaction (RT-PCR)

The mRNA expression was analyzed according to the previous literatures (Zhang H. L. et al., 2022). Total RNA was extracted from tibias using TRIzol (Invitrogen, Carlsbad, CA, United States). The RNA sample was reverse transcribed using a TOBOBlue qRT Premix with a gDNA Eraser 2.0 kit (#RTQ202, Toroivd Technology Co. Ltd., Shanghai, China). The levels of prostaglandin endoperoxide synthase 2 (PTGS2, GenePharma, Shanghai, China) were measured in a RT-PCR (7,500, Applied Biosystems, Waltham, MA, United States) using SYBR Green reagents (#QPS201, Toyobo Co. Ltd., Osaka, Japan). GAPDH was used as a housekeeping gene. The following primer sequences were available: PTGS2 (mouse)-forward: 5′-TAC​CCT​CCT​CAC​ATC​CCT​GA-3′; PTGS2 (mouse)-reversed: 5′-CCT​GCT​TGA​GTA​TGT​CGC​AC-3′; GAPDH (mouse)-forward: 5′-CCA​CCC​AGA​AGA​CTG​TGG​AT-3′; GAPDH (mouse)-reverse: 5′- GGA​TGC​AGG​GAT​GAT​GTT​CT-3′. Data are shown as the fold change relative to controls.

### 2.8 Malondialdehyde (MDA) and GSH measurements

MDA levels in bone were measured using an MDA assay kit (#A003-1-1, Nanjing Jiancheng Bioengineering Institute, Jiangsu, China). MDA contents were detected at 532 nm on a microplate reader (Spectra MAX 190, Molecular Devices, Silicon Valley, CA, United States), and normalized by total protein concentration, which was analyzed using the Bradford method (#KGPBCA, KeyGen, Nanjing, China). GSH content was measured according to the method provided by the GSH and glutathione disulfide (GSSG) assay kit (#S0053, Beyotime, Shanghai, China). The absorbance of each well was measured at a wavelength of 412 nm on a microplate reader (Spectra MAX 190).

### 2.9 Metabolomics analysis

The bone sample (100 mg) was mixed with 500 μL of methanol. The mixture was homogenized and sonicated. Then, 500 μL of CHCl_3_ and 200 μL of water were added to the obtained mixture (Hu et al., 2020). After centrifugation, the bottom organic layer was transferred and lyophilized. The residue was redissolved in 200 μL 10% acetonitrile. The levels of PUFAs in bone samples were determined by UPLC-MS system (ACQUITY UPLC H-Class PLUS, Waters) with an electrospray negative ionization source. The supernatant was separated on a C18 column (ACQUITY UPLC BEH C18, 2.1 × 100 mm, 1.7 μm, Waters). The mobile phase consisted of water (A) and acetonitrile (B) at a flow rate of 0.3 mL/min. The analysis was carried out with an elution gradient as follows: 0–1 min, 10%–50% B; 1–4 min, 50%–80% B; 4–15 min, 80%–100% B. PUFAs were detected using the multiple reaction monitoring (Supplementary Table S2) mode and quantified based on the respective standard curves (Supplementary Table S3). MS data were processed using Masslynx V4.1 software (Waters, MA, United States).

### 2.10 Statistical analysis

Statistical analyses were conducted using GraphPad Prism 9.0 (GraphPad, CA, US). Data are presented as mean ± standard deviation (SD). Statistical comparisons were performed using one-way or two-way analysis of variance followed by Dunnett corrections to compare multiple groups. *p* < 0.05 was considered statistically significant (Huang and Wang, 2022).

## 3 Results

### 3.1 AA/PC attenuated osteoporosis in OVX-induced mice

**FIGURE 1:**
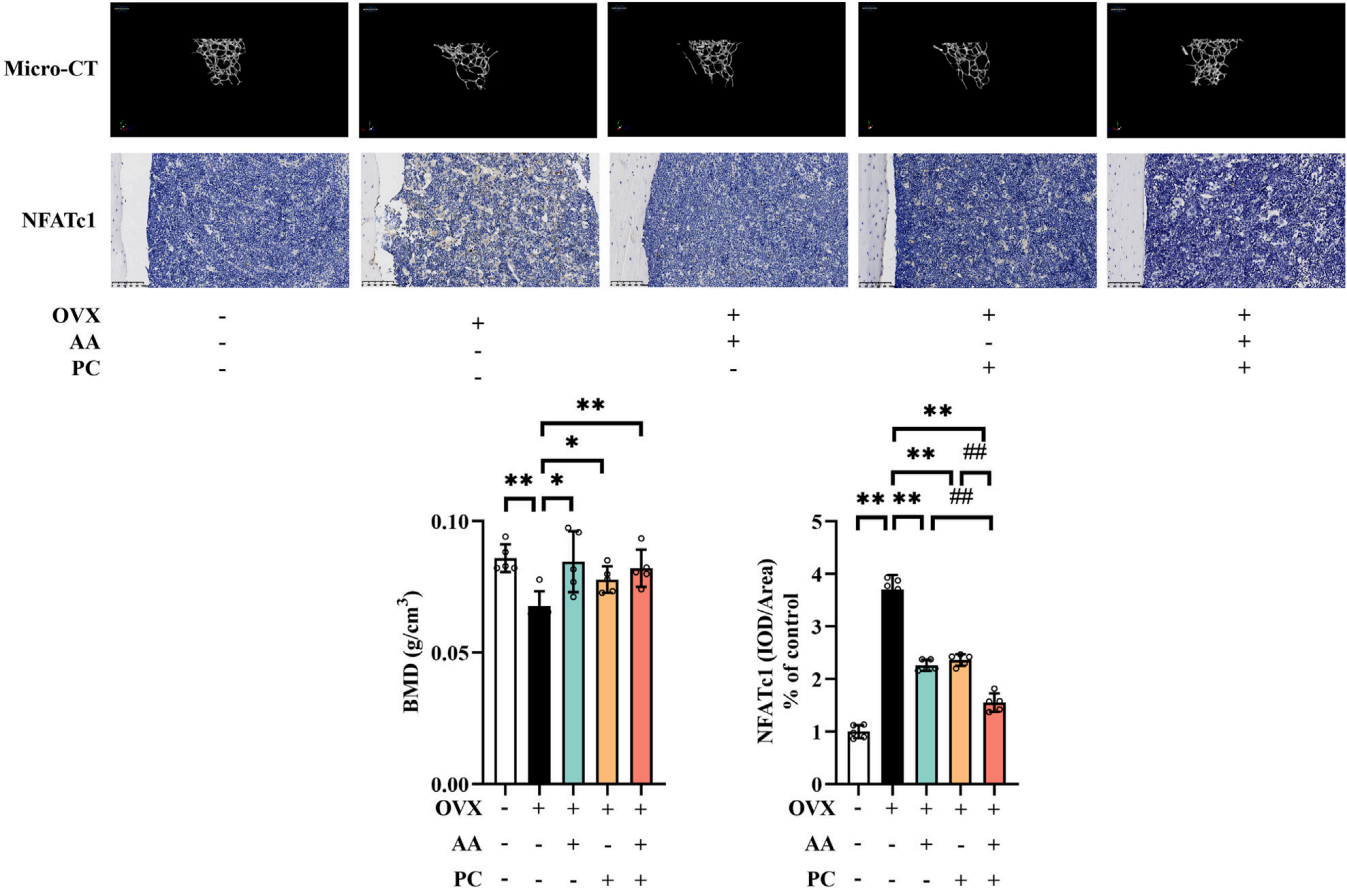
The AA/PC herb pair had a protective effect on osteoporosis caused by OVX *in vivo*. Micro-CT images showing the microarchitecture of the distal femur. IHC analysis of NFATc1 in the femur. Data are expressed as the mean ± SD (n = 5). *p < 0.05, **p < 0.01 compared with the OVX group. ##*p* < 0.01 compared with the AA/PC group.

OVX-induced murine models are commonly used for postmenopausal osteoporosis research (Tao et al., 2021). Herein, OVX induced serious deterioration of bone microstructure (Figure 1) and the Mod group had significantly lower BMD values than did the Sham group (*p* < 0.01). Treatments with AA (*p* < 0.05), PC (*p* < 0.05) and AA/PC (*p* < 0.01) increased BMD in OVX mice.

NFATc1 plays a key role in regulating osteoclast-specific genes and participates in the osteoclast differentiation (Zeng et al., 2016). IHC results showed that NFATc1 level increased in the Mod group (Figure 1B, *p* < 0.01). Administration of AA (*p* < 0.01), PC (*p* < 0.01), and AA/PC (*p* < 0.01) decreased the NFATc1 expression compared with the Mod group. Importantly, AA/PC had a better effect on inhibiting NFATc1 compared with AA (*p* < 0.01) or PC (*p* < 0.01) alone. These results indicated that AA and PC synergistically attenuated osteoporosis in OVX mice.

### 3.2 Serum pharmacochemistry and network pharmacology analysis

To identify the potential therapeutic substances of AA/PC, UPLC-QTOF-MS was used to analyze the serum collected after AA/PC administration. A total of five prototype compounds were identified in the serum (Supplementary Table S1): mangiferin, magnoflorine, berberine, timosaponin BIII, and timosaponin AIII. The extracted ion chromatograms in the positive-ion mode are shown in Supplementary Figure S2.

**FIGURE 2:**
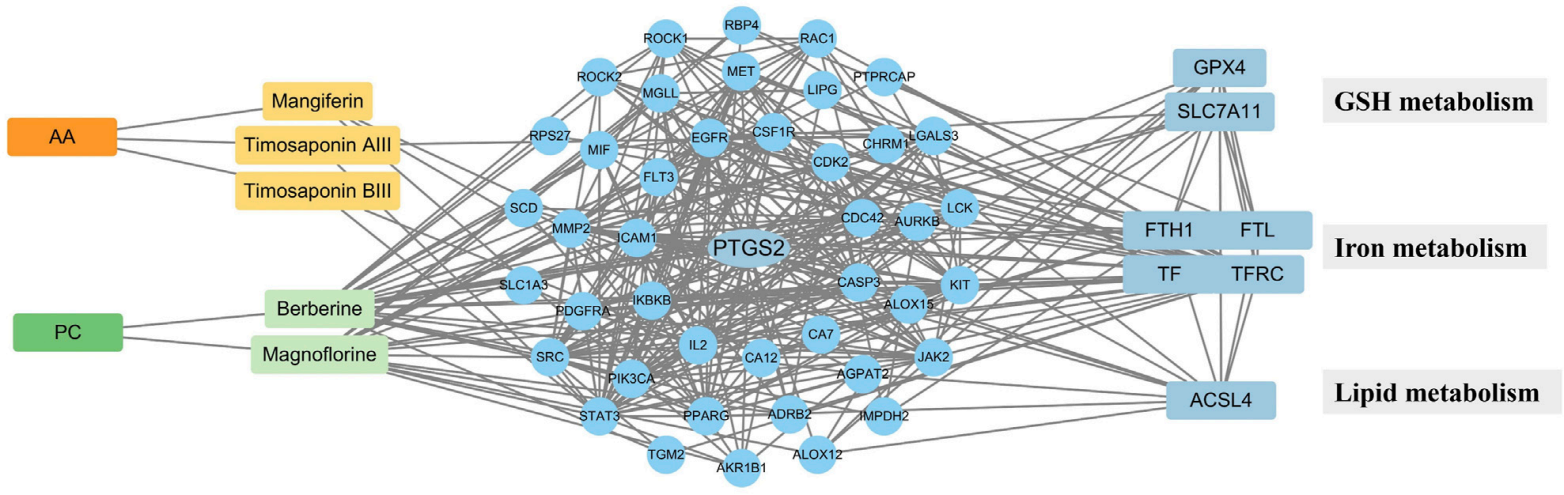
PPI network construction of key targets. The “component-target- metabolism” network.

Ferroptosis often occurs with postmenopausal osteoporosis (Hu Y. et al., 2022). Several compounds from AA/PC also showed anti-ferroptosis effects (Yi et al., 2021; Zhou et al., 2023). The intersection analysis of targets of absorbable components and genes of ferroptosis were then conducted (Figure 2), which included 57 nodes and 352 edges. Among them, PTGS2 had a higher degree (27) and was considered one of the most crucial targets for the absorbable components of AA/PC. The compound target network of each absorbable component, top 10 KEGG pathways, and top 10 enriched GO enrichment terms are shown in Supplementary Figure S3. Collectively, these results suggested that the effect of AA/PC was closely related to ferroptosis pathways.

### 3.3 AA/PC suppressed ferroptosis

**FIGURE 3:**
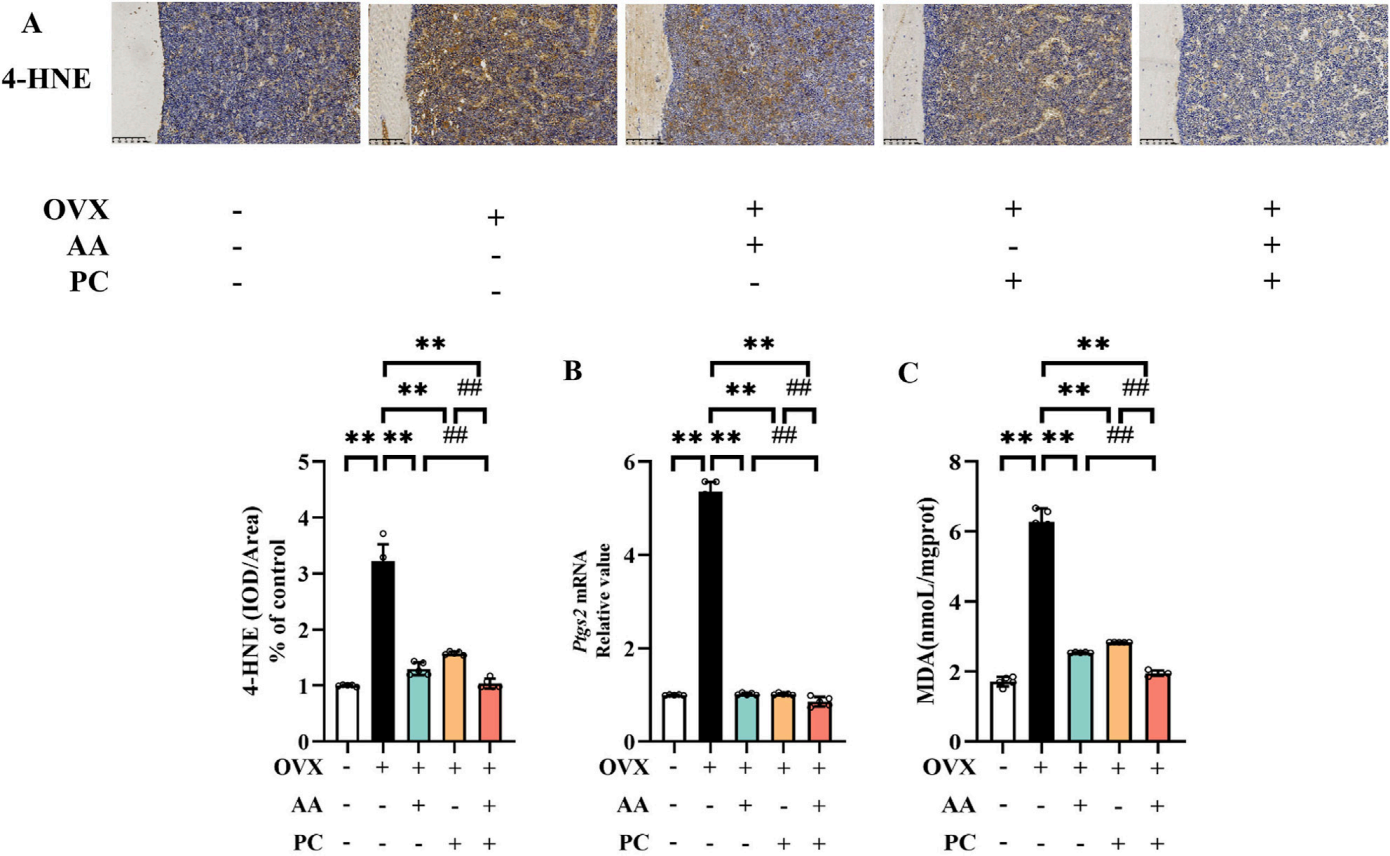
AA and PC exerted synergistic effects on regulating ferroptosis. **(A)** IHC analysis of 4-HNE in the femur. **(B)** The mRNA level of *Ptgs2* in tibias. **(C)** The level of MDA in tibias. Data are expressed as the mean ± SD (n = 5). **p < 0.01 compared with the OVX group. ##*p* < 0.01 compared with the AA/PC group.

Lipid peroxides 4-HNE was detected to explore whether AA/PC suppressed lipid peroxidation. IHC analysis showed that OVX induced a significant increase of 4-HNE in the mouse femur (Figure 3A, *p* < 0.01), while AA (*p* < 0.01), PC (*p* < 0.01), and AA/PC (*p* < 0.01) effectively decreased 4-HNE levels. Interestingly, AA/PC showed the best 4-HNE suppression performance.

PTGS2 is a well-established regulator of ferroptosis. Our results showed that the mRNA of PTGS2 increased in the Mod group (Figure 3B, *p* < 0.01) compared with the Sham group, while AA (*p* < 0.01), PC (*p* < 0.01), and AA/PC (*p* < 0.01) decreased the mRNA levels of PTGS2 in the OVX mouse. Compared with the AA and PC groups, the AA/PC group had lower PTGS2 expression (*p* < 0.01). Additionally, the lipid peroxidation marker MDA in the mouse tibia was detected. OVX increased the MDA levels (Figure 3C, *p* < 0.01), but AA (*p* < 0.01), PC (*p* < 0.01), and AA/PC (*p* < 0.01) treatments decreased MDA levels.

### 3.4 AA/PC activated the SLC7A11/GSH/GPX4 pathway

**FIGURE 4:**
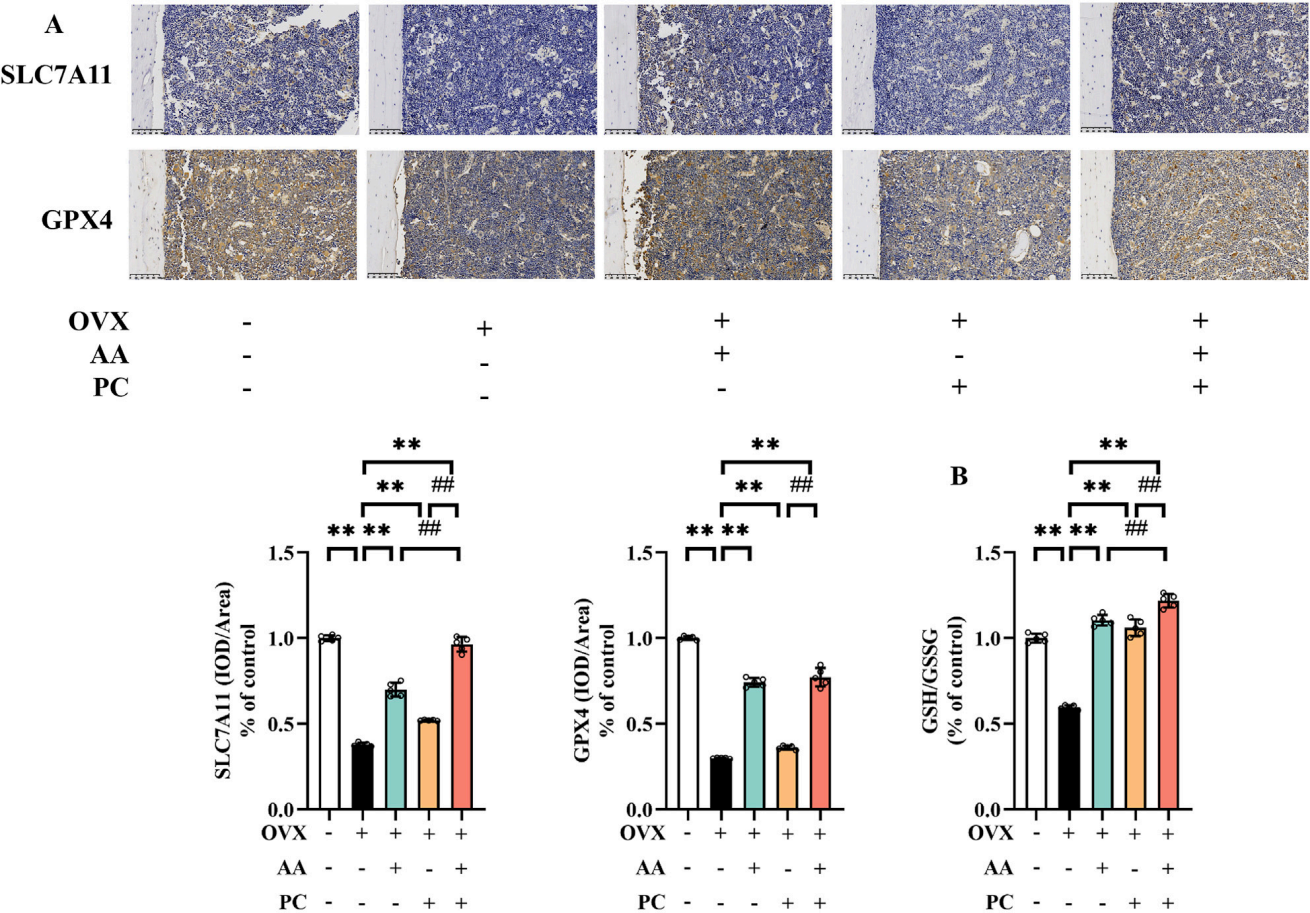
AA and PC exerted synergistic effects on regulating GSH metabolism. **(A)** IHC analysis of SLC7A11 and GPX4 in the femur. **(B)** GSH/GSSG ratio in tibias. Data are expressed as the mean ± SD (n = 5). **p < 0.01 compared with the OVX group. ##*p* < 0.01 compared with the AA/PC group.

SLC7A11/GSH/GPX4 pathway is the canonical ferroptosis-suppressing signaling pathway (Yang et al., 2022). IHC demonstrated that SLC7A11 and GPX4 reduced in the Mod group compared with the Sham group (Figure 4A, *p* < 0.01). Meanwhile, OVX resulted in the decrease of GSH/GSSG ratio (Figure 4B, *p* < 0.01). AA, PC, and AA/PC treatments reversed the expression of SLC7A11 (*p* < 0.01), GPX4 (*p* < 0.01), and GSH/GSSG (*p* < 0.01). AA/PC also had better performance on the stimulation of SLC7A11/GSH/GPX4 pathway compared with the AA or PC groups (*p* < 0.01).

### 3.5 AA/PC regulated the TFRC/ferritin pathway

**FIGURE 5:**
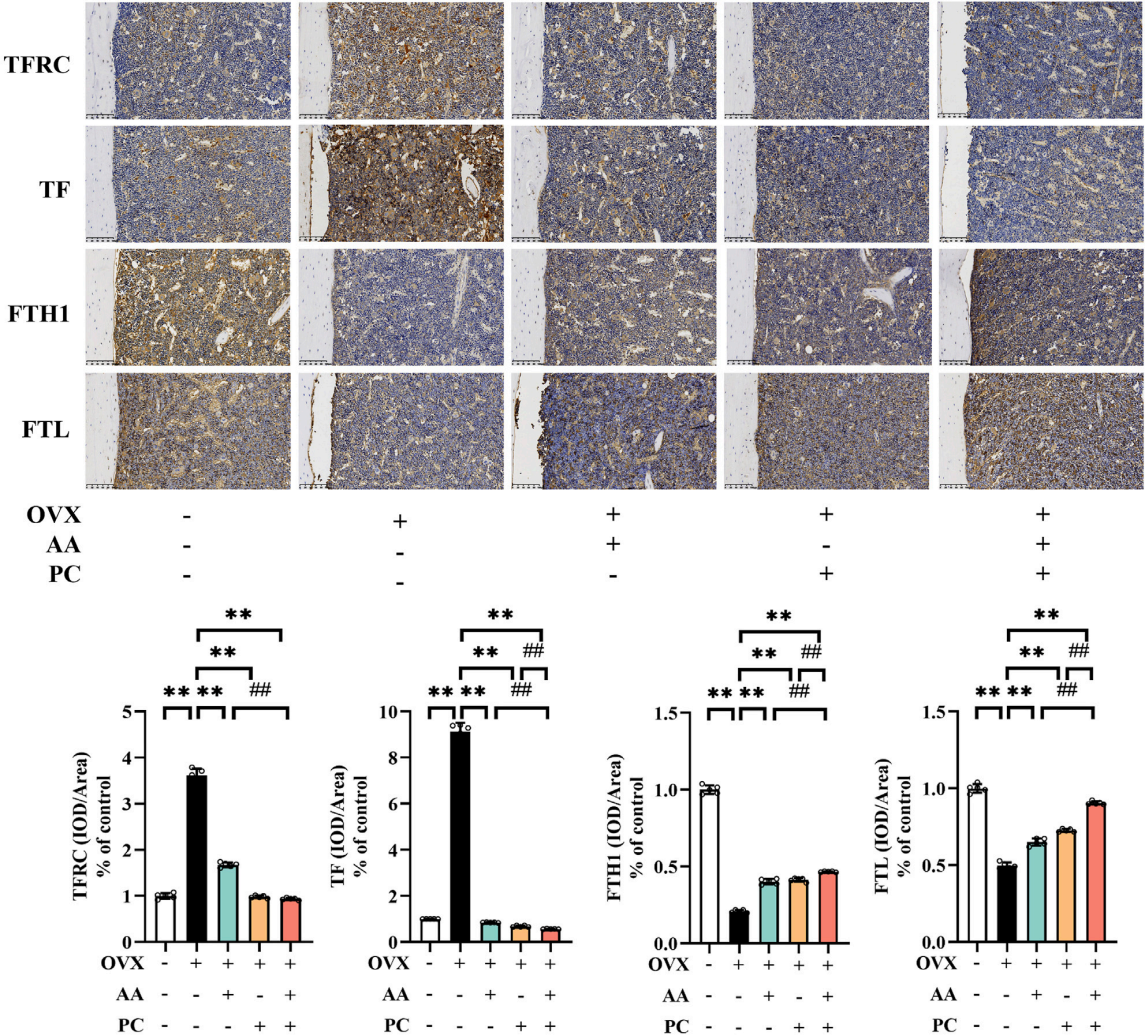
AA and PC exerted synergistic effects on regulating iron metabolism. IHC analysis of TFRC, TF, FTH1, and FTL in the femur. Data are expressed as the mean ± SD (n = 5). **p < 0.01 compared with the OVX group. ##*p* < 0.01 compared with the AA/PC group.

Next, we investigated the expression of iron homeostasis-related markers TFRC, TF, FTH1, and FTL. Protein levels of TFRC and TF were upregulated (Figure 5, *p* < 0.01), while expressions of FTH1 and FTL were downregulated in the Mod group (*p* < 0.01). AA, PC, and AA/PC decreased the protein expression of TFRC (*p* < 0.01) and TF (*p* < 0.01), but increased the levels of FTH1 (*p* < 0.01) and FTL (*p* < 0.01) compared with those of the Mod group (*p* < 0.01). Compared with the single herb groups, the AA/PC group had lower expression of TFRC (*p* < 0.01 for AA) and TF (*p* < 0.01), but higher expression of FTH1 (*p* < 0.01) and FTL (*p* < 0.01). These results showed synergistic effects on the regulation of these iron homeostasis-related markers.

### 3.6 AA/PC mediated lipid peroxidation

**FIGURE 6:**
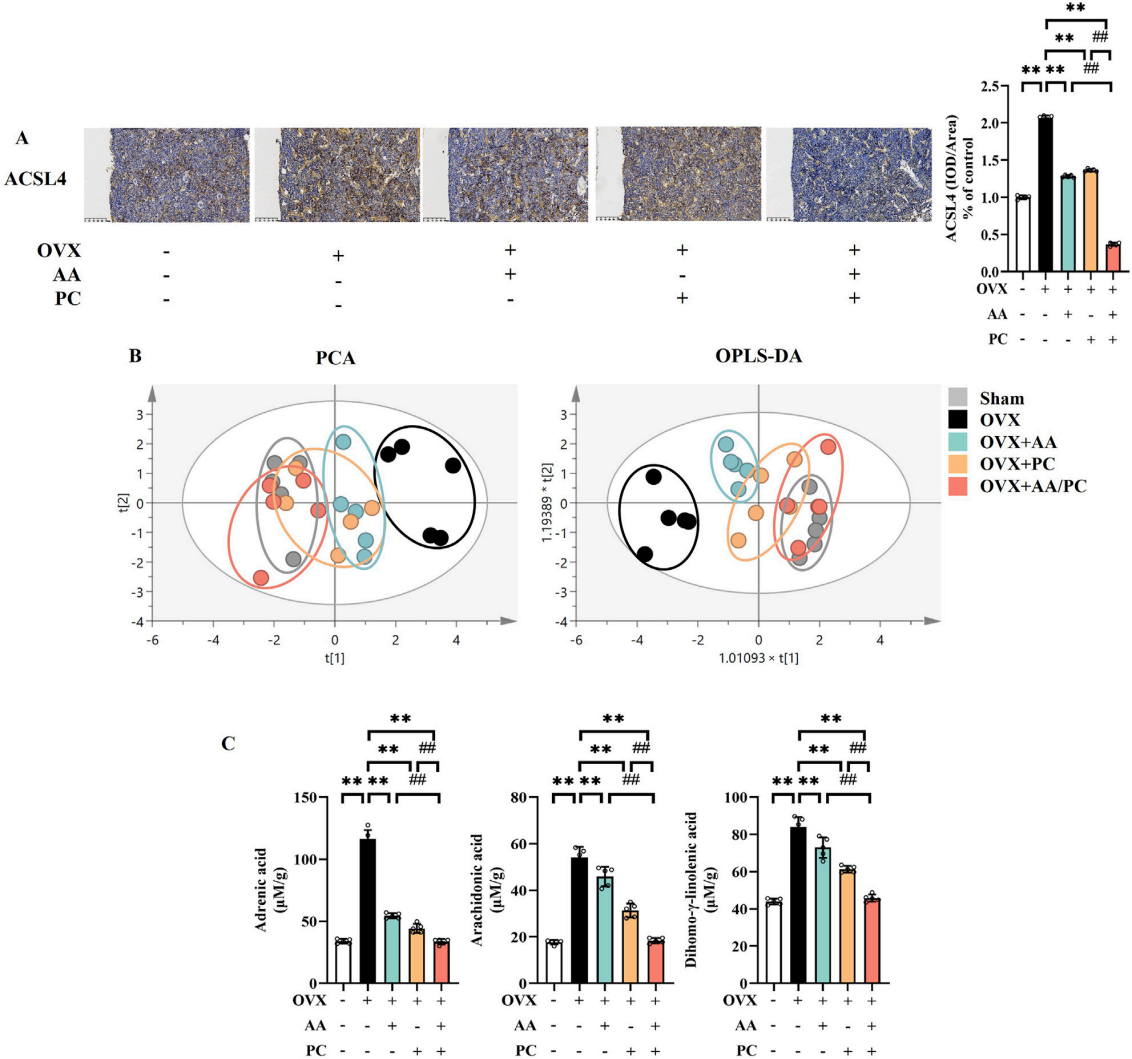
AA and PC exerted synergistic effects on regulating lipid metabolism. **(A)** IHC analysis of ACSL4 in the femur. **(B)** The plot depicts separation of PCA and OPLS-DA of tibias from different groups. **(C)** Targeted metabolomic analyses of tibias were performed using UPLC-MS to measure the concentration of adrenic acid, arachidonic acid, and dihomo-γ-linolenic acid. Data are expressed as the mean ± SD (n = 5). **p < 0.01 compared with the OVX group. ##*p* < 0.01 compared with the AA/PC group.

ACSL4 is an important isozyme for PUFAs metabolism, which dictates ferroptosis sensitivity (Cui et al., 2021). IHC results showed that the protein levels of ACSL4 were upregulated in the Mod group compared with the Sham group (Figure 6A, *p* < 0.01), while treatments of AA, PC, and AA/PC restored these changes (*p* < 0.01). AA/PC showed stronger inhibitory effects on ACSL4 expression compared with AA (*p* < 0.01) or PC (*p* < 0.01) alone.

Metabolomics analysis was carried out to investigate the mechanism underlying the effect of AA/PC on osteoporosis. We compared the contents of individual PUFAs in the tibia from different groups. Targeted metabolomics data showed that the AA/PC groups were closer to the Sham group in both principal component analysis (PCA) (Figure 6B) and orthogonal partial least-squares discriminant analysis (OPLS-DA). These data indicated that the PUFAs metabolic profiles were reversed by AA/PC treatment. Meanwhile, the contents of adrenic acid (ADA) (Figure 6C, *p* < 0.01), arachidonic acid (ARA) (*p* < 0.01), and dihomo-γ-linolenic acid (DGLA, *p* < 0.01) in the Mod group were higher than those in the Sham group. AA/PC reduced ADA (*p* < 0.01), AA (*p* < 0.01), and DGLA (*p* < 0.01) compared with those in the Mod group. Other PUFAs are shown in Supplementary Figure S4.

## 4 Discussion

The herb pair AA/PC provides promising therapeutic benefits for osteoporosis treatment (Xu et al., 2022b). However, the effects of AA/PC on ferroptosis remains unclear. To better understand the pharmacological mechanism of AA/PC, we used an integrated strategy that combined serum pharmacochemistry, network pharmacology analysis, and *in vivo* experimental validations. Our results indicate that AA/PC has synergistic effects on regulating GSH, iron, and lipid metabolism, subsequently inhibiting ferroptosis in bone and attenuating postmenopausal osteoporosis.

The femur is a high-risk area for pathological fractures caused by osteoporosis, and is commonly used to detect the anti-osteoporosis activity of drugs (Blain et al., 2008; Zhang et al., 2024). Therefore, this study mainly focused on the femur of OVX mice. Besides, postmenopausal women are the main population for osteoporosis. Ferroptosis acts as one of the critical pathogenic factors in postmenopausal osteoporosis. Clinical studies have shown that serum ferritin levels in postmenopausal women increase by 2–3 times (Kim C. et al., 2012). Natural compounds show potential as therapeutics for postmenopausal osteoporosis by intervening in ferroptosis. Aconine can regulate osteoclast ferroptosis by suppressing GPX4 and upregulating ACSL4 (Xue et al., 2023). Fructus *Ligustri Lucidi* significantly suppresses ferroptosis, protects osteogenic ability, and promotes the Nrf2/HO-1 signaling pathway (Li et al., 2023). Our work aims to screen the most promising gene targets and pathways for the treatment of postmenopausal osteoporosis with AA/PC.

Herein, AA/PC significantly improved BMD values and bone microarchitecture in OVX-induced mice. Postmenopausal osteoporosis is characterized by elevated bone resorption activity. Osteoclasts are responsible for this, and suppressing osteoclast overactivity has been shown to serve as an effective therapy in clinical treatment (Li et al., 2022). Interestingly, AA and PC had synergistic effects on inhibiting the master regulator of osteoclastogenesis, NFATc1. According to serum pharmacology, active components are initially absorbed into the blood, after which they can regulate pathological processes (Mahana et al., 2023). Herein, five compounds were detected in the serum of OVX mice after AA/PC administration. Recent findings and our previous work have shown that mangiferin from AA, and berberine and magnoflorine from PC, suppress osteoclast differentiation (Sun et al., 2020; Han and Kim, 2019). Although the effects of timosaponin BIII and timosaponin AIII on osteoclastogenesis are not well understood, studies have reported that this kind of steroidal saponin protects osteoblasts and promotes bone formation *in vitro* and *in vivo* (Wang et al., 2020). Our results herein suggest that the therapeutic effects of AA/PC might be attributed to these absorbable components. Subsequent network pharmacology revealed the combined mechanisms of these absorbable components on ferroptosis. We discovered that unique compounds interact with multiple targets, and participate in the supervision of multiple targets. For example: 3 targets in mangiferin, 37 in timosaponin AIII, 4 in timosaponin BIII, 98 in berberine, and 99 in magnoflorine. This indicates that these compounds may have complementary and synergistic therapeutic effects in treating osteoporosis.

Oxidative stress and lipid peroxidation caused by estrogen deficiency are regarded the main causes of elevated bone resorption activity. Herein, AA and PC suppressed lipid peroxidation in OVX mice, evidenced by downregulation of 4-HNE, PTGS2, and MDA. GSH is among the most important cellular antioxidants, and acts as a crucial cofactor for GPX4. SLC7A11 controls the intracellular cystine for GSH synthesis (Ursini and Maiorino, 2020). Our previous work revealed that activation of the SLC7A11/GPX4 signaling pathway inhibits the onset of ferroptosis, and eventually attenuates osteoporosis (Xu et al., 2022a). Among absorbable components in AA, mangiferin has acted as a ferroptosis inhibitor through the Keap1/Nrf2/SLC7A11/GPX4 pathway in osteoporosis mice (Deng et al., 2024). Our work herein showed, for the first time, that AA/PC, especially AA, can upregulate expressions of SLC7A11 and GPX4, as well as the GSH/GSSG ratio, in the bone of OVX mice. Interestingly, we found AA/PC had better performance for activating the SLC7A11/GSH/GPX4 pathway than AA or PC alone.

ASCL4 upregulation also contributes to lipid peroxidation (Zhang Z. et al., 2022). PUFA-containing phospholipids are the main substrates of lipid peroxidation in ferroptosis, which is positively regulated by ACSL4 (Liu et al., 2022a). According to network pharmacology analysis, both AA and PC may regulate ACSL4. *In vivo* experiments confirmed that AA and PC could reduce the expression of ACSL4 in OVX mice. AA and PC showed a synergistic inhibition effect on ACSL4. Furthermore, we conducted a lipid profiling analysis using UPLC-MS/MS. ARA and ADA are the main substrates of lipid peroxidation in ferroptosis (Tang et al., 2021). Our results show that the contents of ARA and ADA increased in the Mod group compared with those in the Sham group, while AA and PC restored this change. DGLA has been observed to trigger ferroptosis (Perez et al., 2020). AA and PC could reduce the contents of DGLA in OVX mice. Consistently, AA/PC showed better regulation effects on the PUFA profile than did the single herb group.

Furthermore, accumulation of intracellular iron promotes lipid peroxidation, leading to ferroptosis (Park and Chung, 2019). TF is an iron-binding serum protein and TFRC induces cellular uptake of iron from TF. FTH1/FTL are intracellular iron storage proteins (Liu et al., 2022b). Previous studies showed that berberine from PC reduced TFRC but increased FTH in imatinib mesylate-induced H9c2 cells and mice (Song et al., 2023). The PPI network herein revealed that the five absorbable components of AA/PC were closely related to the TFRC/ferritin pathway. The pharmacological experiments further confirmed that AA/PC, especially PC, rescued the TFRC/ferritin pathway in the bone of OVX mice. Interestingly, we that found AA/PC had better performance on activating the TFRC/ferritin pathway than did AA or PC alone.

## 5 Conclusion

Our cumulative findings indicate that the herb pair AA/PC reduced bone resorption and alleviated osteoporosis in OVX mice. By integrating serum pharmacochemistry and network pharmacology, five absorbable components of AA/PC were found to contribute to the inhibitory effects on ferroptosis. *In vivo* experiments showed that AA and PC synergistically activated the SLC7A11/GSH/GPX4 pathway, suppressed ACSL4, and inhibited the TFRC/ferritin pathway. Our findings reveal a combined mechanism of the AA/PC herb pair for postmenopausal osteoporosis treatment, and provide a rational approach for clarifying the composition rules of traditional Chinese medicine.

## Data Availability

The original contributions presented in the study are included in the article/Supplementary Material, further inquiries can be directed to the corresponding author.
