# Supplementary material for: Synergistic anti-osteoporosis effects of Anemarrhena asphodeloides bunge–Phellodendron chinense C.K. Schneid herb pair via ferroptosis suppression in ovariectomized mice
Source: Front Pharmacol. 2024 Oct 24;15:1378634. doi: 10.3389/fphar.2024.1378634 (PMC11540766; doi:10.3389/fphar.2024.1378634)
Supplement: Supplementary file 1 [file DataSheet1.docx]

Supplementary Material

## Supplementary Figures


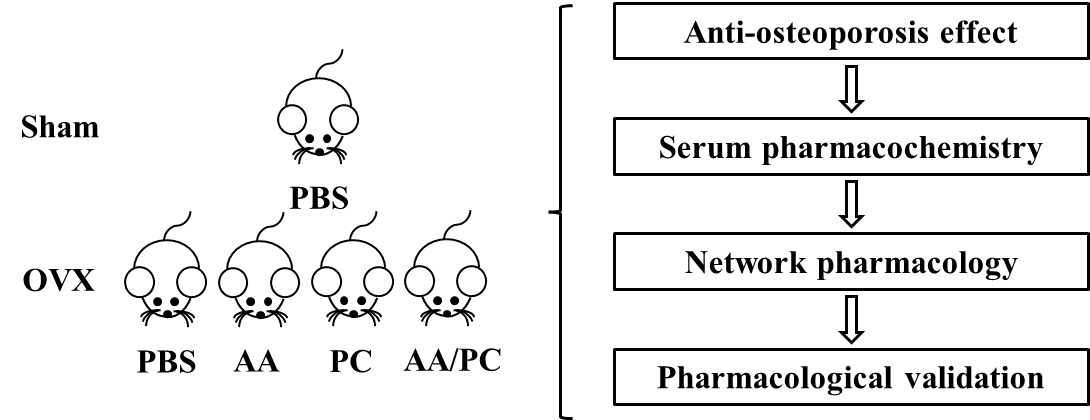


**Figure S1.** Experimental diagram.


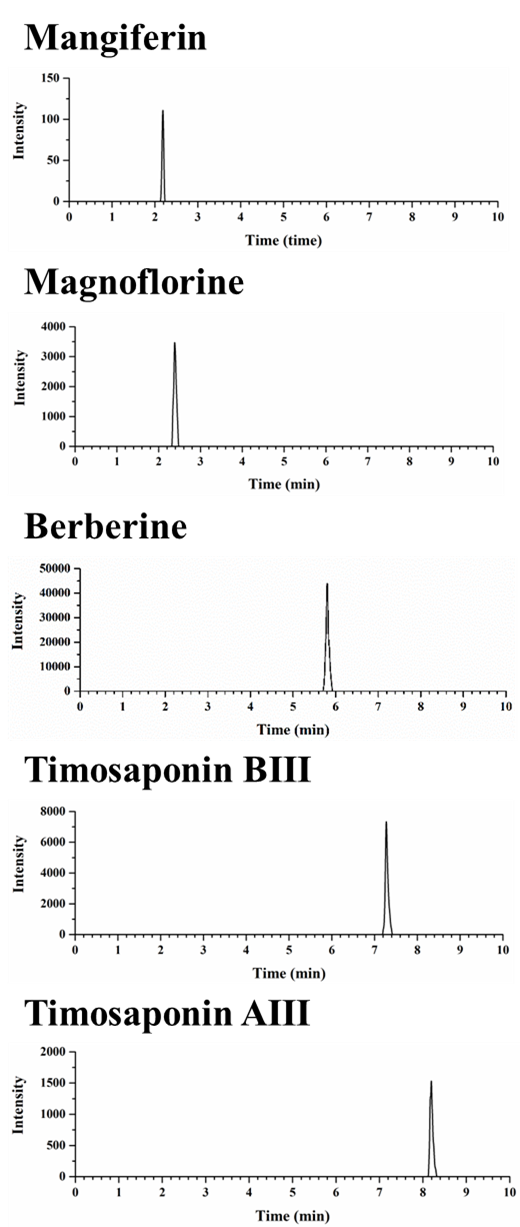


**Figure S2.** Extracted ion chromatograms for mangiferin, magnoflorine, berberine, timosaponin BⅢ and timosaponin AⅢ.


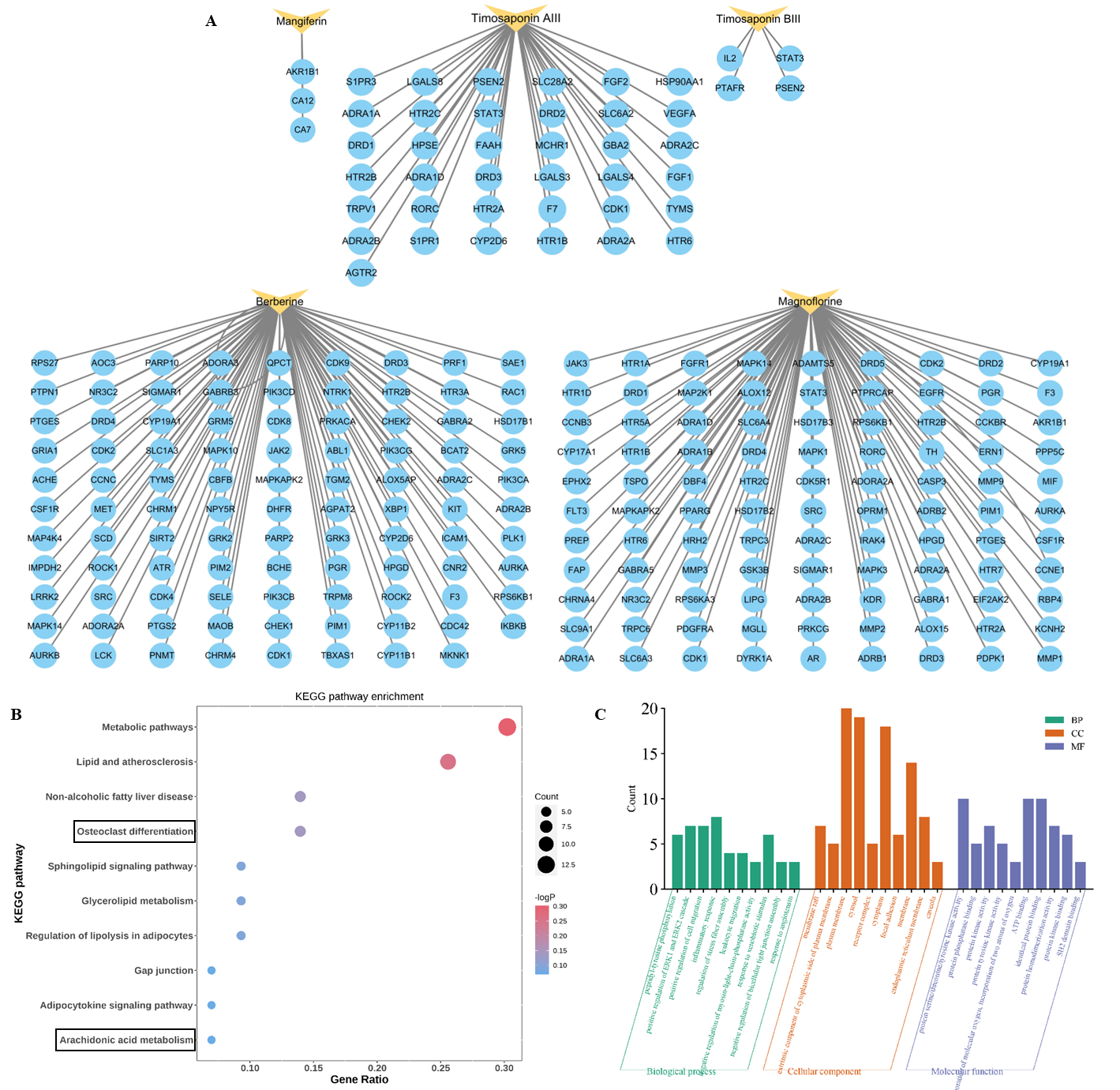


**Figure S3.** (A) Compound target network of each absorbable component. (B) Top 10 enriched KEGG pathways of AA/PC targets. (C) Molecular function analysis, biological process analysis, and cellular component analysis for AA/PC targets.


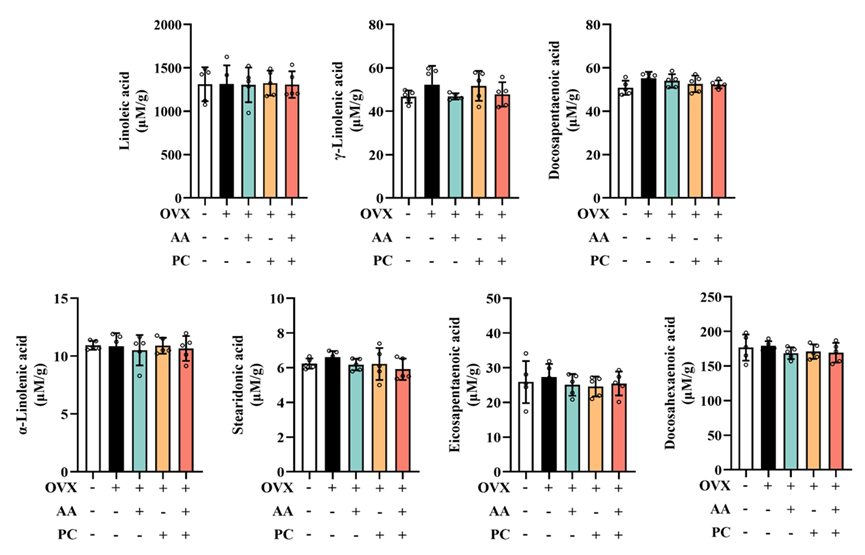


**Figure S4** Targeted metabolomic analyses of tibias were performed using UPLC-MS to measure the concentration of individual PUFA.

## Supplementary Tables

**Table 1**. Prototype compounds identified in serum after oral administration of AA/PC.

| NO. | t_R_/min | Identification | Formula | Precursor ion | Selective ion | Fragment ions (m/z) |
| --- | --- | --- | --- | --- | --- | --- |
| 1  2  3  4  5 | 2.21  2.38  5.8  7.27  8.18 | Mangiferin  Magnoflorine  Berberine  Timosaponin BⅢ  Timosaponin AⅢ | C_19_H_18_O_11_  C_20_H_24_NO_4_  C_20_H_18_NO_4_  C_45_H_74_O_18_  C_39_H_64_O_13_ | 423.0922  342.1700  336.123  903.4948  741.4420 | [M+H]^+^  [M]^+^  [M]^+^  [M+H]^+^  [M+H]^+^ | 303 [M+H-C_4_H_8_O_4_]^+^, 273 [M+H-C_4_H_8_O_4_-CH_2_O]^+^  265 [M-C_2_H_7_N-CH_3_OH]^+^  320 [M-CH_4_]^+^, 292 [M-CH_4_-CO]^+^  741 [M+H-Gcl]^+^, 579 [M+H-2Gcl]^+^, 417 [M+H-2Gcl-Gal]^+^  579 [M+H-Gcl]^+^ |

**Table S2.** MRM of PUFAs analysis.

| Compound | Transition | Cone | Collision |
| --- | --- | --- | --- |
| Adrenic acid | 331.3 > 331.3 | 40 | 20 |
| Docosapentaenoic acid | 329.2 > 329.2 | 48 | 20 |
| Docosahexaenoic acid  Dihomo-γ-linolenic acid  Arachidonic acid  Eicosapentaenoic acid  Linoleic acid  α-Linolenic acid  γ-Linolenic acid  Stearidonic acid | 327.4 > 327.4  305.4 > 305.4  303.2 > 303.2  301.2 > 301.2  279.3 > 279.3  277.22 > 277.56  277.2 > 277.2  275.3 > 275.3 | 46  46  46  46  46  54  46  36 | 16  14  18  16  16  10  22  16 |

**Table S3.** Linear equation of PUFAs in tibia bone.

| Compound | Linear Equation | R^2^ |
| --- | --- | --- |
| Adrenic acid | y = 225x + 67 | 0.9991 |
| Docosapentaenoic acid | y = 219.54x - 218.17 | 0.999 |
| Docosahexaenoic acid | y = 338.09x + 44.54 | 0.998 |
| Dihomo-γ-linolenic acid  Arachidonic acid  Eicosapentaenoic acid  Linoleic acid  α-Linolenic acid  γ-Linolenic acid  Stearidonic acid | y = 1846.3x - 1649.9  y = 5166x - 3548.7  y = 260.27x + 291.71  y = 384.24x - 15.423  y = 19423x - 14964  y = 51.95x - 21.883  y = 271.34x - 134.13 | 1  1  0.9998  0.9989  0.9995  0.9996  0.9995 |
